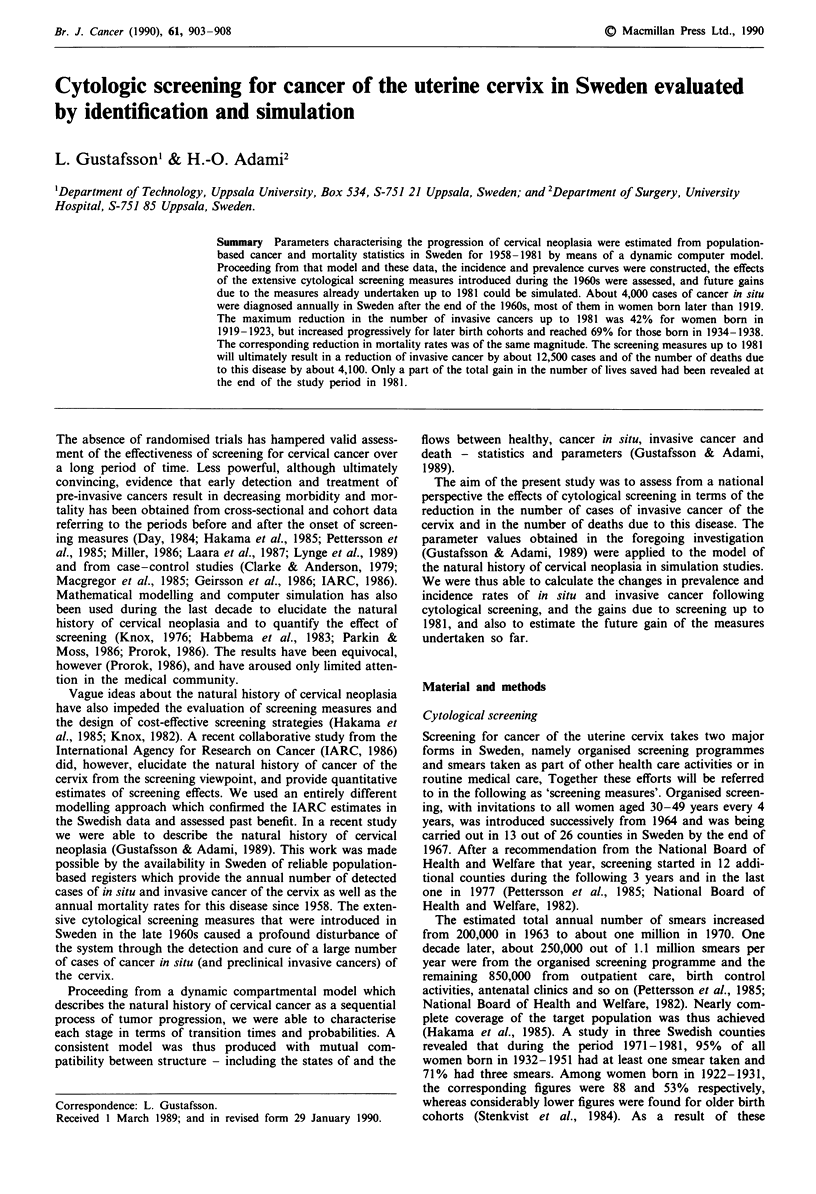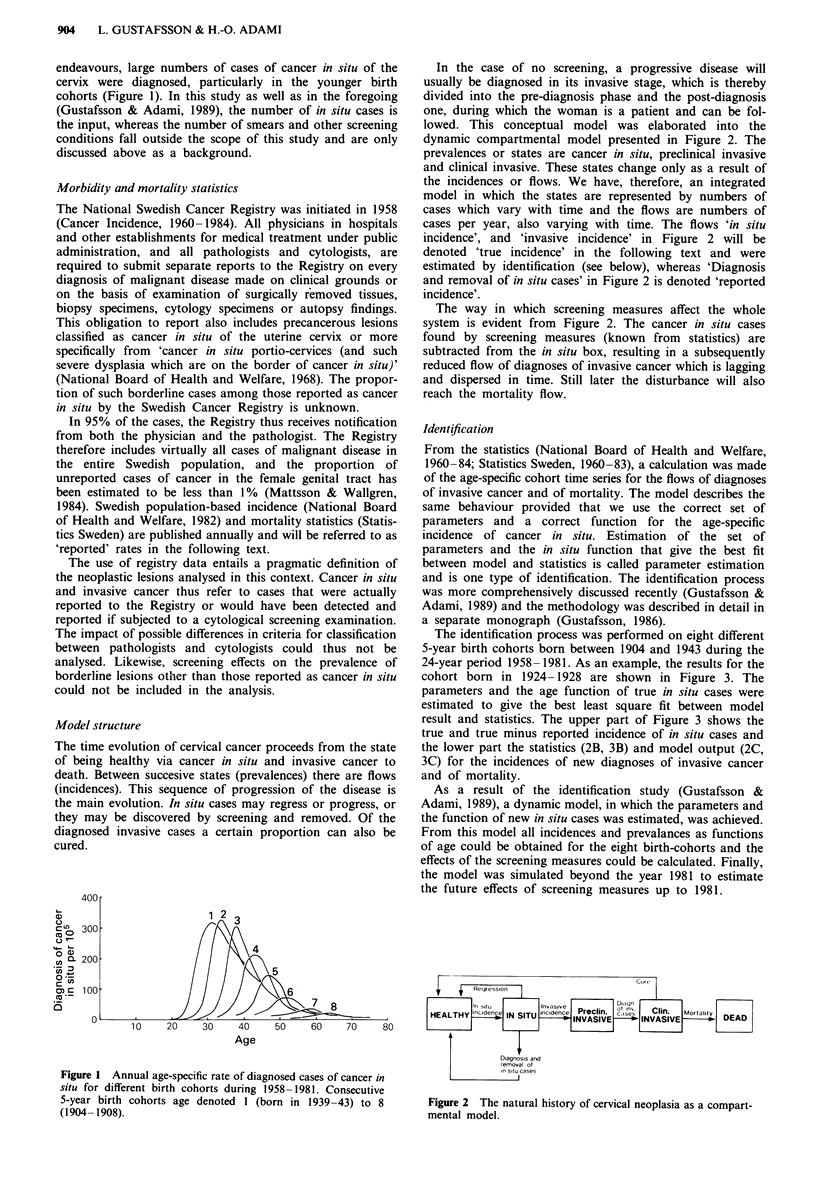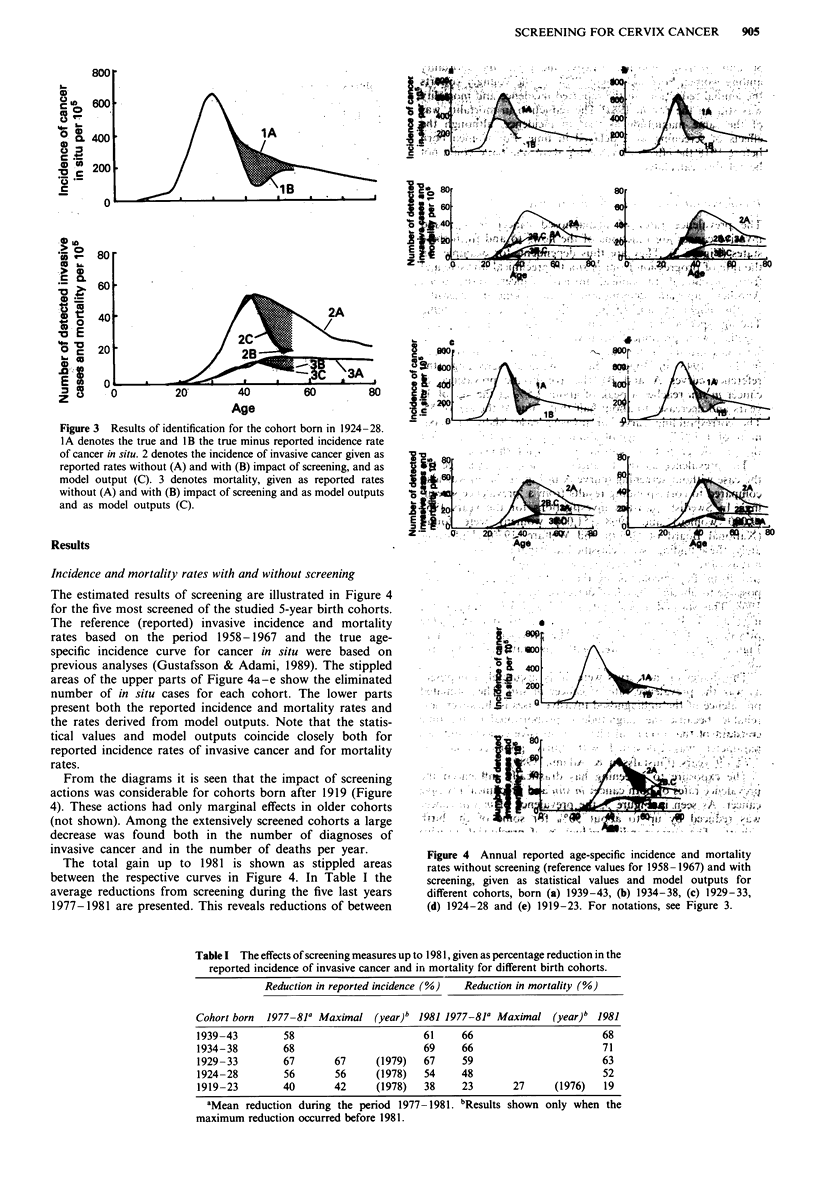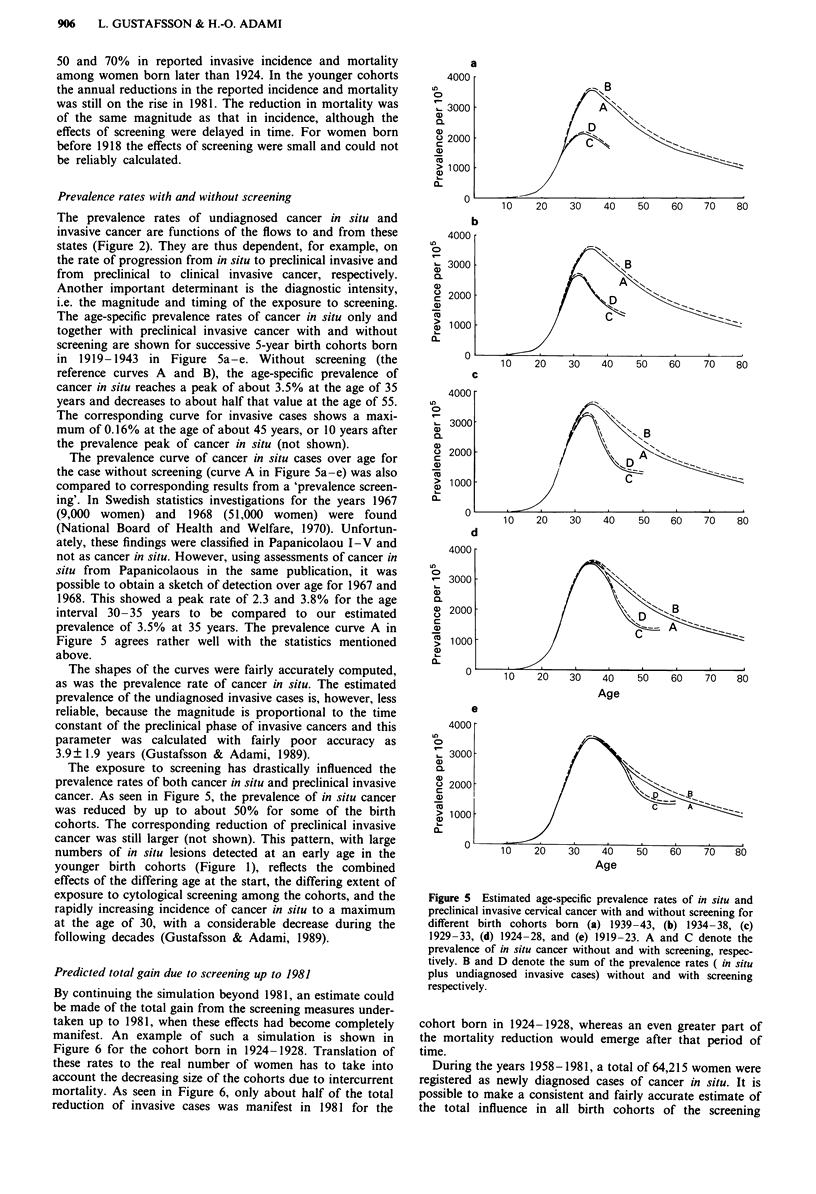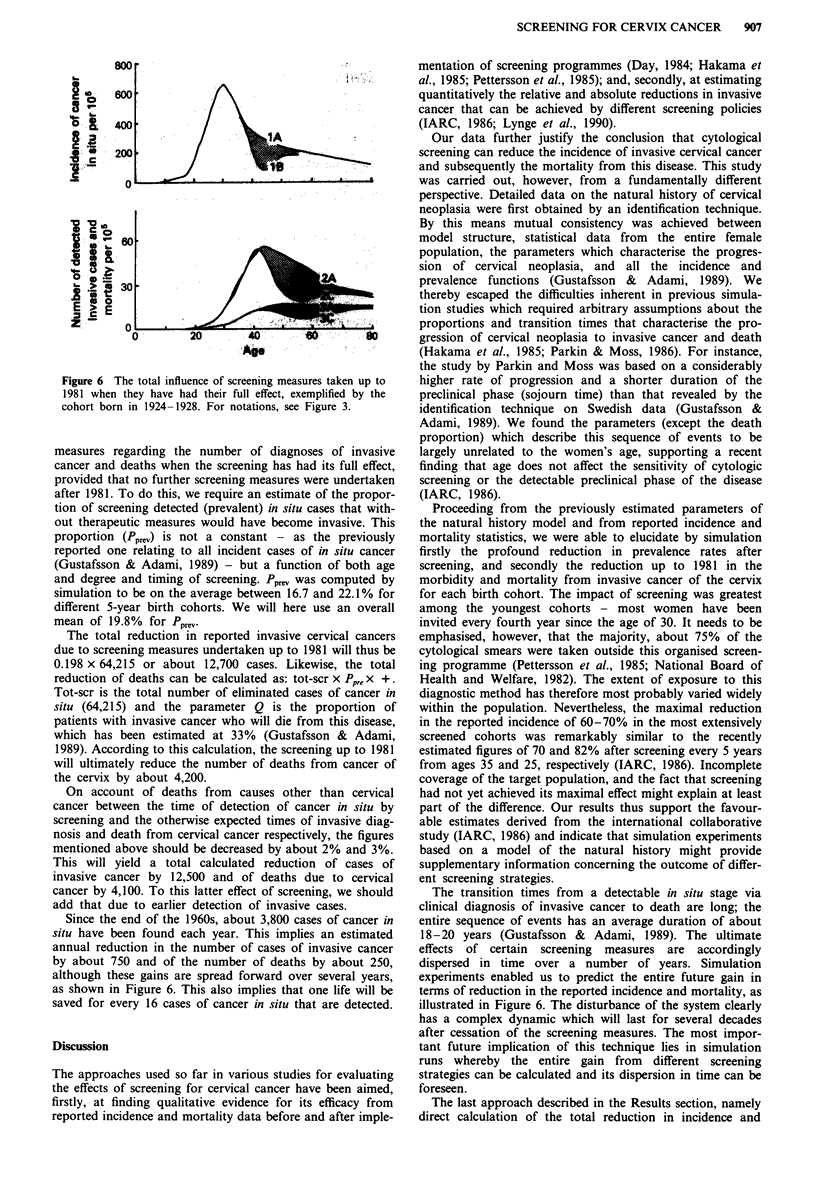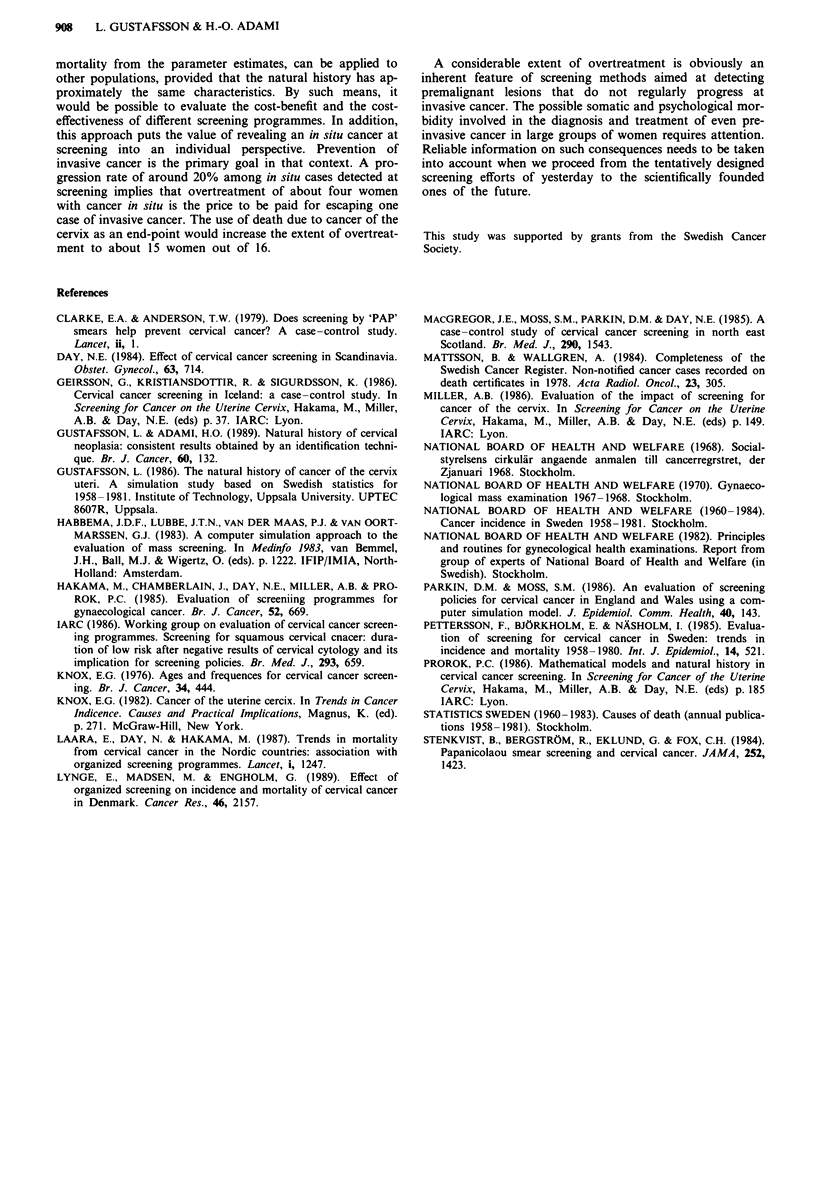# Cytologic screening for cancer of the uterine cervix in Sweden evaluated by identification and simulation.

**DOI:** 10.1038/bjc.1990.202

**Published:** 1990-06

**Authors:** L. Gustafsson, H. O. Adami

**Affiliations:** Department of Technology, Uppsala University, Sweden.

## Abstract

Parameters characterising the progression of cervical neoplasia were estimated from population-based cancer and mortality statistics in Sweden for 1958-1981 by means of a dynamic computer model. Proceeding from that model and these data, the incidence and prevalence curves were constructed, the effects of the extensive cytological screening measures introduced during the 1960s were assessed, and future gains due to the measures already undertaken up to 1981 could be simulated. About 4,000 cases of cancer in situ were diagnosed annually in Sweden after the end of the 1960s, most of them in women born later than 1919. The maximum reduction in the number of invasive cancers up to 1981 was 42% for women born in 1919-1923, but increased progressively for later birth cohorts and reached 69% for those born in 1934-1938. The corresponding reduction in mortality rates was of the same magnitude. The screening measures up to 1981 will ultimately result in a reduction of invasive cancer by about 12,500 cases and of the number of deaths due to this disease by about 4,100. Only a part of the total gain in the number of lives saved had been revealed at the end of the study period in 1981.


Br. J. Cancer (1990), 61, 903-908 © Macmillan Press Ltd., 1990

### Cytologic screening for cancer of the uterine cervix in Sweden evaluated by identification and simulation

L. Gustafsson[1] & H.-O. Adami[2]

[1]*Department of Technology, Uppsala University, Box 534, S-751 21 Uppsala, Sweden; and* [2]*Department of Surgery, University Hospital, S-751 85 Uppsala, Sweden.*

**Summary** Parameters characterising the progression of cervical neoplasia were estimated from population-based cancer and mortality statistics in Sweden for 1958–1981 by means of a dynamic computer model. Proceeding from that model and these data, the incidence and prevalence curves were constructed, the effects of the extensive cytological screening measures introduced during the 1960s were assessed, and future gains due to the measures already undertaken up to 1981 could be simulated. About 4,000 cases of cancer *in situ* were diagnosed annually in Sweden after the end of the 1960s, most of them in women born later than 1919. The maximum reduction in the number of invasive cancers up to 1981 was 42% for women born in 1919–1923, but increased progressively for later birth cohorts and reached 69% for those born in 1934–1938. The corresponding reduction in mortality rates was of the same magnitude. The screening measures up to 1981 will ultimately result in a reduction of invasive cancer by about 12,500 cases and of the number of deaths due to this disease by about 4,100. Only a part of the total gain in the number of lives saved had been revealed at the end of the study period in 1981.

The absence of randomised trials has hampered valid assessment of the effectiveness of screening for cervical cancer over a long period of time. Less powerful, although ultimately convincing, evidence that early detection and treatment of pre-invasive cancers result in decreasing morbidity and mortality has been obtained from cross-sectional and cohort data referring to the periods before and after the onset of screening measures (Day, 1984; Hakama *et al.*, 1985; Pettersson *et al.*, 1985; Miller, 1986; Laara *et al.*, 1987; Lynge *et al.*, 1989) and from case–control studies (Clarke & Anderson, 1979; Macgregor *et al.*, 1985; Geirsson *et al.*, 1986; IARC, 1986). Mathematical modelling and computer simulation has also been used during the last decade to elucidate the natural history of cervical neoplasia and to quantify the effect of screening (Knox, 1976; Habbema *et al.*, 1983; Parkin & Moss, 1986; Prorok, 1986). The results have been equivocal, however (Prorok, 1986), and have aroused only limited attention in the medical community.

Vague ideas about the natural history of cervical neoplasia have also impeded the evaluation of screening measures and the design of cost-effective screening strategies (Hakama *et al.*, 1985; Knox, 1982). A recent collaborative study from the International Agency for Research on Cancer (IARC, 1986) did, however, elucidate the natural history of cancer of the cervix from the screening viewpoint, and provide quantitative estimates of screening effects. We used an entirely different modelling approach which confirmed the IARC estimates in the Swedish data and assessed past benefit. In a recent study we were able to describe the natural history of cervical neoplasia (Gustafsson & Adami, 1989). This work was made possible by the availability in Sweden of reliable population-based registers which provide the annual number of detected cases of *in situ* and invasive cancer of the cervix as well as the annual mortality rates for this disease since 1958. The extensive cytological screening measures that were introduced in Sweden in the late 1960s caused a profound disturbance of the system through the detection and cure of a large number of cases of cancer *in situ* (and preclinical invasive cancers) of the cervix.

Proceeding from a dynamic compartmental model which describes the natural history of cervical cancer as a sequential process of tumor progression, we were able to characterise each stage in terms of transition times and probabilities. A consistent model was thus produced with mutual compatibility between structure – including the states of and the flows between healthy, cancer *in situ*, invasive cancer and death – statistics and parameters (Gustafsson & Adami, 1989).

The aim of the present study was to assess from a national perspective the effects of cytological screening in terms of the reduction in the number of cases of invasive cancer of the cervix and in the number of deaths due to this disease. The parameter values obtained in the foregoing investigation (Gustafsson & Adami, 1989) were applied to the model of the natural history of cervical neoplasia in simulation studies. We were thus able to calculate the changes in prevalence and incidence rates of *in situ* and invasive cancer following cytological screening, and the gains due to screening up to 1981, and also to estimate the future gain of the measures undertaken so far.

### Material and methods

### *Cytological screening*

Screening for cancer of the uterine cervix takes two major forms in Sweden, namely organised screening programmes and smears taken as part of other health care activities or in routine medical care, Together these efforts will be referred to in the following as 'screening measures'. Organised screening, with invitations to all women aged 30–49 years every 4 years, was introduced successively from 1964 and was being carried out in 13 out of 26 counties in Sweden by the end of 1967. After a recommendation from the National Board of Health and Welfare that year, screening started in 12 additional counties during the following 3 years and in the last one in 1977 (Pettersson *et al.*, 1985; National Board of Health and Welfare, 1982).

The estimated total annual number of smears increased from 200,000 in 1963 to about one million in 1970. One decade later, about 250,000 out of 1.1 million smears per year were from the organised screening programme and the remaining 850,000 from outpatient care, birth control activities, antenatal clinics and so on (Pettersson *et al.*, 1985; National Board of Health and Welfare, 1982). Nearly complete coverage of the target population was thus achieved (Hakama *et al.*, 1985). A study in three Swedish counties revealed that during the period 1971–1981, 95% of all women born in 1932–1951 had at least one smear taken and 71% had three smears. Among women born in 1922–1931, the corresponding figures were 88 and 53% respectively, whereas considerably lower figures were found for older birth cohorts (Stenkvist *et al.*, 1984). As a result of these

Correspondence: L. Gustafsson.
Received 1 March 1989; and in revised form 29 January 1990.

endeavours, large numbers of cases of cancer *in situ* of the cervix were diagnosed, particularly in the younger birth cohorts (Figure 1). In this study as well as in the foregoing (Gustafsson & Adami, 1989), the number of *in situ* cases is the input, whereas the number of smears and other screening conditions fall outside the scope of this study and are only discussed above as a background.

*Morbidity and mortality statistics*

The National Swedish Cancer Registry was initiated in 1958 (Cancer Incidence, 1960–1984). All physicians in hospitals and other establishments for medical treatment under public administration, and all pathologists and cytologists, are required to submit separate reports to the Registry on every diagnosis of malignant disease made on clinical grounds or on the basis of examination of surgically removed tissues, biopsy specimens, cytology specimens or autopsy findings. This obligation to report also includes precancerous lesions classified as cancer *in situ* of the uterine cervix or more specifically from 'cancer *in situ* portio-cervices (and such severe dysplasia which are on the border of cancer *in situ)*' (National Board of Health and Welfare, 1968). The proportion of such borderline cases among those reported as cancer *in situ* by the Swedish Cancer Registry is unknown.

In 95% of the cases, the Registry thus receives notification from both the physician and the pathologist. The Registry therefore includes virtually all cases of malignant disease in the entire Swedish population, and the proportion of unreported cases of cancer in the female genital tract has been estimated to be less than 1% (Mattsson & Wallgren, 1984). Swedish population-based incidence (National Board of Health and Welfare, 1982) and mortality statistics (Statistics Sweden) are published annually and will be referred to as 'reported' rates in the following text.

The use of registry data entails a pragmatic definition of the neoplastic lesions analysed in this context. Cancer *in situ* and invasive cancer thus refer to cases that were actually reported to the Registry or would have been detected and reported if subjected to a cytological screening examination. The impact of possible differences in criteria for classification between pathologists and cytologists could thus not be analysed. Likewise, screening effects on the prevalence of borderline lesions other than those reported as cancer *in situ* could not be included in the analysis.

*Model structure*

The time evolution of cervical cancer proceeds from the state of being healthy via cancer *in situ* and invasive cancer to death. Between succesive states (prevalences) there are flows (incidences). This sequence of progression of the disease is the main evolution. *In situ* cases may regress or progress, or they may be discovered by screening and removed. Of the diagnosed invasive cases a certain proportion can also be cured.

**Figure 1** Annual age-specific rate of diagnosed cases of cancer *in situ* for different birth cohorts during 1958–1981. Consecutive 5-year birth cohorts age denoted 1 (born in 1939–43) to 8 (1904–1908).

In the case of no screening, a progressive disease will usually be diagnosed in its invasive stage, which is thereby divided into the pre-diagnosis phase and the post-diagnosis one, during which the woman is a patient and can be followed. This conceptual model was elaborated into the dynamic compartmental model presented in Figure 2. The prevalences or states are cancer *in situ*, preclinical invasive and clinical invasive. These states change only as a result of the incidences or flows. We have, therefore, an integrated model in which the states are represented by numbers of cases which vary with time and the flows are numbers of cases per year, also varying with time. The flows '*in situ* incidence', and 'invasive incidence' in Figure 2 will be denoted 'true incidence' in the following text and were estimated by identification (see below), whereas 'Diagnosis and removal of *in situ* cases' in Figure 2 is denoted 'reported incidence'.

The way in which screening measures affect the whole system is evident from Figure 2. The cancer *in situ* cases found by screening measures (known from statistics) are subtracted from the *in situ* box, resulting in a subsequently reduced flow of diagnoses of invasive cancer which is lagging and dispersed in time. Still later the disturbance will also reach the mortality flow.

*Identification*

From the statistics (National Board of Health and Welfare, 1960–84; Statistics Sweden, 1960–83), a calculation was made of the age-specific cohort time series for the flows of diagnoses of invasive cancer and of mortality. The model describes the same behaviour provided that we use the correct set of parameters and a correct function for the age-specific incidence of cancer *in situ*. Estimation of the set of parameters and the *in situ* function that give the best fit between model and statistics is called parameter estimation and is one type of identification. The identification process was more comprehensively discussed recently (Gustafsson & Adami, 1989) and the methodology was described in detail in a separate monograph (Gustafsson, 1986).

The identification process was performed on eight different 5-year birth cohorts born between 1904 and 1943 during the 24-year period 1958–1981. As an example, the results for the cohort born in 1924–1928 are shown in Figure 3. The parameters and the age function of true *in situ* cases were estimated to give the best least square fit between model result and statistics. The upper part of Figure 3 shows the true and true minus reported incidence of *in situ* cases and the lower part the statistics (2B, 3B) and model output (2C, 3C) for the incidences of new diagnoses of invasive cancer and of mortality.

As a result of the identification study (Gustafsson & Adami, 1989), a dynamic model, in which the parameters and the function of new *in situ* cases was estimated, was achieved. From this model all incidences and prevalances as functions of age could be obtained for the eight birth-cohorts and the effects of the screening measures could be calculated. Finally, the model was simulated beyond the year 1981 to estimate the future effects of screening measures up to 1981.

**Figure 2** The natural history of cervical neoplasia as a compartmental model.

**Figure 3** Results of identification for the cohort born in 1924–28. 1A denotes the true and 1B the true minus reported incidence rate of cancer *in situ*. 2 denotes the incidence of invasive cancer given as reported rates without (A) and with (B) impact of screening, and as model output (C). 3 denotes mortality, given as reported rates without (A) and with (B) impact of screening and as model outputs and as model outputs (C).

### Results

### *Incidence and mortality rates with and without screening*

The estimated results of screening are illustrated in Figure 4 for the five most screened of the studied 5-year birth cohorts. The reference (reported) invasive incidence and mortality rates based on the period 1958–1967 and the true age-specific incidence curve for cancer *in situ* were based on previous analyses (Gustafsson & Adami, 1989). The stippled areas of the upper parts of Figure 4a–e show the eliminated number of *in situ* cases for each cohort. The lower parts present both the reported incidence and mortality rates and the rates derived from model outputs. Note that the statistical values and model outputs coincide closely both for reported incidence rates of invasive cancer and for mortality rates.

From the diagrams it is seen that the impact of screening actions was considerable for cohorts born after 1919 (Figure 4). These actions had only marginal effects in older cohorts (not shown). Among the extensively screened cohorts a large decrease was found both in the number of diagnoses of invasive cancer and in the number of deaths per year.

The total gain up to 1981 is shown as stippled areas between the respective curves in Figure 4. In Table I the average reductions from screening during the five last years 1977–1981 are presented. This reveals reductions of between

**Figure 4** Annual reported age-specific incidence and mortality rates without screening (reference values for 1958–1967) and with screening, given as statistical values and model outputs for different cohorts, born (**a**) 1939–43, (**b**) 1934–38, (**c**) 1929–33, (**d**) 1924–28 and (**e**) 1919–23. For notations, see Figure 3.

**Table I** The effects of screening measures up to 1981, given as percentage reduction in the reported incidence of invasive cancer and in mortality for different birth cohorts.

| | *Reduction in reported incidence (%)* | | | | *Reduction in mortality (%)* | | | |
|---|---|---|---|---|---|---|---|---|
| *Cohort born* | *1977–81*[a] | *Maximal* | *(year)*[b] | *1981* | *1977–81*[a] | *Maximal* | *(year)*[b] | *1981* |
| 1939–43 | 58 | | | 61 | 66 | | | 68 |
| 1934–38 | 68 | | | 69 | 66 | | | 71 |
| 1929–33 | 67 | 67 | (1979) | 67 | 59 | | | 63 |
| 1924–28 | 56 | 56 | (1978) | 54 | 48 | | | 52 |
| 1919–23 | 40 | 42 | (1978) | 38 | 23 | 27 | (1976) | 19 |

[a]Mean reduction during the period 1977–1981. [b]Results shown only when the maximum reduction occurred before 1981.

50 and 70% in reported invasive incidence and mortality among women born later than 1924. In the younger cohorts the annual reductions in the reported incidence and mortality was still on the rise in 1981. The reduction in mortality was of the same magnitude as that in incidence, although the effects of screening were delayed in time. For women born before 1918 the effects of screening were small and could not be reliably calculated.

### *Prevalence rates with and without screening*

The prevalence rates of undiagnosed cancer *in situ* and invasive cancer are functions of the flows to and from these states (Figure 2). They are thus dependent, for example, on the rate of progression from *in situ* to preclinical invasive and from preclinical to clinical invasive cancer, respectively. Another important determinant is the diagnostic intensity, i.e. the magnitude and timing of the exposure to screening. The age-specific prevalence rates of cancer *in situ* only and together with preclinical invasive cancer with and without screening are shown for successive 5-year birth cohorts born in 1919–1943 in Figure 5a–e. Without screening (the reference curves A and B), the age-specific prevalence of cancer *in situ* reaches a peak of about 3.5% at the age of 35 years and decreases to about half that value at the age of 55. The corresponding curve for invasive cases shows a maximum of 0.16% at the age of about 45 years, or 10 years after the prevalence peak of cancer *in situ* (not shown).

The prevalence curve of cancer *in situ* cases over age for the case without screening (curve A in Figure 5a–e) was also compared to corresponding results from a 'prevalence screening'. In Swedish statistics investigations for the years 1967 (9,000 women) and 1968 (51,000 women) were found (National Board of Health and Welfare, 1970). Unfortunately, these findings were classified in Papanicolaou I–V and not as cancer *in situ*. However, using assessments of cancer *in situ* from Papanicolaous in the same publication, it was possible to obtain a sketch of detection over age for 1967 and 1968. This showed a peak rate of 2.3 and 3.8% for the age interval 30–35 years to be compared to our estimated prevalence of 3.5% at 35 years. The prevalence curve A in Figure 5 agrees rather well with the statistics mentioned above.

The shapes of the curves were fairly accurately computed, as was the prevalence rate of cancer *in situ*. The estimated prevalence of the undiagnosed invasive cases is, however, less reliable, because the magnitude is proportional to the time constant of the preclinical phase of invasive cancers and this parameter was calculated with fairly poor accuracy as 3.9±1.9 years (Gustafsson & Adami, 1989).

The exposure to screening has drastically influenced the prevalence rates of both cancer *in situ* and preclinical invasive cancer. As seen in Figure 5, the prevalence of *in situ* cancer was reduced by up to about 50% for some of the birth cohorts. The corresponding reduction of preclinical invasive cancer was still larger (not shown). This pattern, with large numbers of *in situ* lesions detected at an early age in the younger birth cohorts (Figure 1), reflects the combined effects of the differing age at the start, the differing extent of exposure to cytological screening among the cohorts, and the rapidly increasing incidence of cancer *in situ* to a maximum at the age of 30, with a considerable decrease during the following decades (Gustafsson & Adami, 1989).

### *Predicted total gain due to screening up to 1981*

By continuing the simulation beyond 1981, an estimate could be made of the total gain from the screening measures undertaken up to 1981, when these effects had become completely manifest. An example of such a simulation is shown in Figure 6 for the cohort born in 1924–1928. Translation of these rates to the real number of women has to take into account the decreasing size of the cohorts due to intercurrent mortality. As seen in Figure 6, only about half of the total reduction of invasive cases was manifest in 1981 for the cohort born in 1924–1928, whereas an even greater part of the mortality reduction would emerge after that period of time.

**Figure 5** Estimated age-specific prevalence rates of *in situ* and preclinical invasive cervical cancer with and without screening for different birth cohorts born (**a**) 1939–43, (**b**) 1934–38, (**c**) 1929–33, (**d**) 1924–28, and (**e**) 1919–23. A and C denote the prevalence of *in situ* cancer without and with screening, respectively. B and D denote the sum of the prevalence rates ( *in situ* plus undiagnosed invasive cases) without and with screening respectively.

During the years 1958–1981, a total of 64,215 women were registered as newly diagnosed cases of cancer *in situ*. It is possible to make a consistent and fairly accurate estimate of the total influence in all birth cohorts of the screening

**Figure 6** The total influence of screening measures taken up to 1981 when they have had their full effect, exemplified by the cohort born in 1924–1928. For notations, see Figure 3.

measures regarding the number of diagnoses of invasive cancer and deaths when the screening has had its full effect, provided that no further screening measures were undertaken after 1981. To do this, we require an estimate of the proportion of screening detected (prevalent) *in situ* cases that without therapeutic measures would have become invasive. This proportion ($P_{prev}$) is not a constant – as the previously reported one relating to all incident cases of *in situ* cancer (Gustafsson & Adami, 1989) – but a function of both age and degree and timing of screening. $P_{prev}$ was computed by simulation to be on the average between 16.7 and 22.1% for different 5-year birth cohorts. We will here use an overall mean of 19.8% for $P_{prev}$.

The total reduction in reported invasive cervical cancers due to screening measures undertaken up to 1981 will thus be $0.198 \times 64{,}215$ or about 12,700 cases. Likewise, the total reduction of deaths can be calculated as: tot-scr $\times P_{pre} \times +$. Tot-scr is the total number of eliminated cases of cancer *in situ* (64,215) and the parameter $Q$ is the proportion of patients with invasive cancer who will die from this disease, which has been estimated at 33% (Gustafsson & Adami, 1989). According to this calculation, the screening up to 1981 will ultimately reduce the number of deaths from cancer of the cervix by about 4,200.

On account of deaths from causes other than cervical cancer between the time of detection of cancer *in situ* by screening and the otherwise expected times of invasive diagnosis and death from cervical cancer respectively, the figures mentioned above should be decreased by about 2% and 3%. This will yield a total calculated reduction of cases of invasive cancer by 12,500 and of deaths due to cervical cancer by 4,100. To this latter effect of screening, we should add that due to earlier detection of invasive cases.

Since the end of the 1960s, about 3,800 cases of cancer *in situ* have been found each year. This implies an estimated annual reduction in the number of cases of invasive cancer by about 750 and of the number of deaths by about 250, although these gains are spread forward over several years, as shown in Figure 6. This also implies that one life will be saved for every 16 cases of cancer *in situ* that are detected.

### Discussion

The approaches used so far in various studies for evaluating the effects of screening for cervical cancer have been aimed, firstly, at finding qualitative evidence for its efficacy from reported incidence and mortality data before and after implementation of screening programmes (Day, 1984; Hakama *et al.*, 1985; Pettersson *et al.*, 1985); and, secondly, at estimating quantitatively the relative and absolute reductions in invasive cancer that can be achieved by different screening policies (IARC, 1986; Lynge *et al.*, 1990).

Our data further justify the conclusion that cytological screening can reduce the incidence of invasive cervical cancer and subsequently the mortality from this disease. This study was carried out, however, from a fundamentally different perspective. Detailed data on the natural history of cervical neoplasia were first obtained by an identification technique. By this means mutual consistency was achieved between model structure, statistical data from the entire female population, the parameters which characterise the progression of cervical neoplasia, and all the incidence and prevalence functions (Gustafsson & Adami, 1989). We thereby escaped the difficulties inherent in previous simulation studies which required arbitrary assumptions about the proportions and transition times that characterise the progression of cervical neoplasia to invasive cancer and death (Hakama *et al.*, 1985; Parkin & Moss, 1986). For instance, the study by Parkin and Moss was based on a considerably higher rate of progression and a shorter duration of the preclinical phase (sojourn time) than that revealed by the identification technique on Swedish data (Gustafsson & Adami, 1989). We found the parameters (except the death proportion) which describe this sequence of events to be largely unrelated to the women's age, supporting a recent finding that age does not affect the sensitivity of cytologic screening or the detectable preclinical phase of the disease (IARC, 1986).

Proceeding from the previously estimated parameters of the natural history model and from reported incidence and mortality statistics, we were able to elucidate by simulation firstly the profound reduction in prevalence rates after screening, and secondly the reduction up to 1981 in the morbidity and mortality from invasive cancer of the cervix for each birth cohort. The impact of screening was greatest among the youngest cohorts – most women have been invited every fourth year since the age of 30. It needs to be emphasised, however, that the majority, about 75% of the cytological smears were taken outside this organised screening programme (Pettersson *et al.*, 1985; National Board of Health and Welfare, 1982). The extent of exposure to this diagnostic method has therefore most probably varied widely within the population. Nevertheless, the maximal reduction in the reported incidence of 60–70% in the most extensively screened cohorts was remarkably similar to the recently estimated figures of 70 and 82% after screening every 5 years from ages 35 and 25, respectively (IARC, 1986). Incomplete coverage of the target population, and the fact that screening had not yet achieved its maximal effect might explain at least part of the difference. Our results thus support the favourable estimates derived from the international collaborative study (IARC, 1986) and indicate that simulation experiments based on a model of the natural history might provide supplementary information concerning the outcome of different screening strategies.

The transition times from a detectable *in situ* stage via clinical diagnosis of invasive cancer to death are long; the entire sequence of events has an average duration of about 18–20 years (Gustafsson & Adami, 1989). The ultimate effects of certain screening measures are accordingly dispersed in time over a number of years. Simulation experiments enabled us to predict the entire future gain in terms of reduction in the reported incidence and mortality, as illustrated in Figure 6. The disturbance of the system clearly has a complex dynamic which will last for several decades after cessation of the screening measures. The most important future implication of this technique lies in simulation runs whereby the entire gain from different screening strategies can be calculated and its dispersion in time can be foreseen.

The last approach described in the Results section, namely direct calculation of the total reduction in incidence and mortality from the parameter estimates, can be applied to other populations, provided that the natural history has approximately the same characteristics. By such means, it would be possible to evaluate the cost-benefit and the cost-effectiveness of different screening programmes. In addition, this approach puts the value of revealing an *in situ* cancer at screening into an individual perspective. Prevention of invasive cancer is the primary goal in that context. A progression rate of around 20% among *in situ* cases detected at screening implies that overtreatment of about four women with cancer *in situ* is the price to be paid for escaping one case of invasive cancer. The use of death due to cancer of the cervix as an end-point would increase the extent of overtreatment to about 15 women out of 16.

A considerable extent of overtreatment is obviously an inherent feature of screening methods aimed at detecting premalignant lesions that do not regularly progress at invasive cancer. The possible somatic and psychological morbidity involved in the diagnosis and treatment of even pre-invasive cancer in large groups of women requires attention. Reliable information on such consequences needs to be taken into account when we proceed from the tentatively designed screening efforts of yesterday to the scientifically founded ones of the future.

This study was supported by grants from the Swedish Cancer Society.

### References

CLARKE, E.A. & ANDERSON, T.W. (1979). Does screening by 'PAP' smears help prevent cervical cancer? A case-control study. *Lancet*, **ii**, 1.

DAY, N.E. (1984). Effect of cervical cancer screening in Scandinavia. *Obstet. Gynecol.*, **63**, 714.

GEIRSSON, G., KRISTIANSDOTTIR, R. & SIGURDSSON, K. (1986). Cervical cancer screening in Iceland: a case-control study. In *Screening for Cancer on the Uterine Cervix*, Hakama, M., Miller, A.B. & Day, N.E. (eds) p. 37. IARC: Lyon.

GUSTAFSSON, L. & ADAMI, H.O. (1989). Natural history of cervical neoplasia: consistent results obtained by an identification technique. *Br. J. Cancer*, **60**, 132.

GUSTAFSSON, L. (1986). The natural history of cancer of the cervix uteri. A simulation study based on Swedish statistics for 1958-1981. Institute of Technology, Uppsala University. UPTEC 8607R, Uppsala.

HABBEMA, J.D.F., LUBBE, J.T.N., VAN DER MAAS, P.J. & VAN OORTMARSSEN, G.J. (1983). A computer simulation approach to the evaluation of mass screening. In *Medinfo 1983*, van Bemmel, J.H., Ball, M.J. & Wigertz, O. (eds). p. 1222. IFIP/IMIA, North-Holland: Amsterdam.

HAKAMA, M., CHAMBERLAIN, J., DAY, N.E., MILLER, A.B. & PROROK, P.C. (1985). Evaluation of screeniing programmes for gynaecological cancer. *Br. J. Cancer*, **52**, 669.

IARC (1986). Working group on evaluation of cervical cancer screening programmes. Screening for squamous cervical cnacer: duration of low risk after negative results of cervical cytology and its implication for screening policies. *Br. Med. J.*, **293**, 659.

KNOX, E.G. (1976). Ages and frequences for cervical cancer screening. *Br. J. Cancer*, **34**, 444.

KNOX, E.G. (1982). Cancer of the uterine cercix. In *Trends in Cancer Indicence. Causes and Practical Implications*, Magnus, K. (ed). p. 271. McGraw-Hill, New York.

LAARA, E., DAY, N. & HAKAMA, M. (1987). Trends in mortality from cervical cancer in the Nordic countries: association with organized screening programmes. *Lancet*, **i**, 1247.

LYNGE, E., MADSEN, M. & ENGHOLM, G. (1989). Effect of organized screening on incidence and mortality of cervical cancer in Denmark. *Cancer Res.*, **46**, 2157.

MACGREGOR, J.E., MOSS, S.M., PARKIN, D.M. & DAY, N.E. (1985). A case-control study of cervical cancer screening in north east Scotland. *Br. Med. J.*, **290**, 1543.

MATTSSON, B. & WALLGREN, A. (1984). Completeness of the Swedish Cancer Register. Non-notified cancer cases recorded on death certificates in 1978. *Acta Radiol. Oncol.*, **23**, 305.

MILLER, A.B. (1986). Evaluation of the impact of screening for cancer of the cervix. In *Screening for Cancer on the Uterine Cervix*, Hakama, M., Miller, A.B. & Day, N.E. (eds) p. 149. IARC: Lyon.

NATIONAL BOARD OF HEALTH AND WELFARE (1968). Socialstyrelsens cirkulär angaende anmalen till cancerregrstret, der Zjanuari 1968. Stockholm.

NATIONAL BOARD OF HEALTH AND WELFARE (1970). Gynaecological mass examination 1967-1968. Stockholm.

NATIONAL BOARD OF HEALTH AND WELFARE (1960-1984). Cancer incidence in Sweden 1958-1981. Stockholm.

NATIONAL BOARD OF HEALTH AND WELFARE (1982). Principles and routines for gynecological health examinations. Report from group of experts of National Board of Health and Welfare (in Swedish). Stockholm.

PARKIN, D.M. & MOSS, S.M. (1986). An evaluation of screening policies for cervical cancer in England and Wales using a computer simulation model. *J. Epidemiol. Comm. Health*, **40**, 143.

PETTERSSON, F., BJÖRKHOLM, E. & NÄSHOLM, I. (1985). Evaluation of screening for cervical cancer in Sweden: trends in incidence and mortality 1958-1980. *Int. J. Epidemiol.*, **14**, 521.

PROROK, P.C. (1986). Mathematical models and natural history in cervical cancer screening. In *Screening for Cancer of the Uterine Cervix*, Hakama, M., Miller, A.B. & Day, N.E. (eds) p. 185 IARC: Lyon.

STATISTICS SWEDEN (1960-1983). Causes of death (annual publications 1958-1981). Stockholm.

STENKVIST, B., BERGSTRÖM, R., EKLUND, G. & FOX, C.H. (1984). Papanicolaou smear screening and cervical cancer. *JAMA*, **252**, 1423.